# Exercise Pulmonary Hypertension and Beyond: Insights in Exercise Pathophysiology in Pulmonary Arterial Hypertension (PAH) from Invasive Cardiopulmonary Exercise Testing

**DOI:** 10.3390/jcm14030804

**Published:** 2025-01-26

**Authors:** Elizabeth S. Tarras, Inderjit Singh, Joan Kreiger, Phillip Joseph

**Affiliations:** Division of Pulmonary, Critical Care and Sleep Medicine, Yale University School of Medicine, New Haven, CT 06511, USA; inderjit.singh@yale.edu (I.S.);

**Keywords:** pulmonary arterial hypertension, pulmonary vascular disease, exercise pulmonary hypertension, invasive cardiopulmonary exercise testing

## Abstract

Pulmonary arterial hypertension (PAH) is a rare, progressive disease of the pulmonary vasculature that is associated with pulmonary vascular remodeling and right heart failure. While there have been recent advances both in understanding pathobiology and in diagnosis and therapeutic options, PAH remains a disease with significant delays in diagnosis and high morbidity and mortality. Information from invasive cardiopulmonary exercise testing (iCPET) presents an important opportunity to evaluate the dynamic interactions within and between the right heart circulatory system and the skeletal muscle during different loading conditions to enhance early diagnosis, phenotype disease subtypes, and personalize treatment in PAH given the shortcomings of contemporary diagnostic and therapeutic approaches. The purpose of this review is to present the current applications of iCPET in PAH and to discuss future applications of the testing methodology.

## 1. Introduction

Pulmonary arterial hypertension (PAH) is a heterogeneous disease of the pulmonary vasculature, characterized by pulmonary vascular remodeling and an increased right ventricular (RV) afterload, which leads to RV maladaptation and right heart failure [1,2,3]. It is a rare disease with an estimated incidence and prevalence of 2.0–5.0 per million adults and 15.0–55.0 per million adults, respectively, and is increasingly being diagnosed in older individuals with cardiovascular comorbidities [4,5,6,7,8,9]. PAH is defined as pre-capillary pulmonary hypertension (PH) with a mean pulmonary arterial pressure (mPAP) >20 mmHg, a pulmonary vascular resistance (PVR) >2 Wood units (WU) and a pulmonary capillary wedge pressure (PAWP) ≤15 mmHg during resting supine right heart catheterization (RHC) [4].

A multiparameter risk stratification approach has reoriented the contemporary PAH treatment strategy, and current guidelines recommend the tailored utilization of therapies that target multiple molecular pathways in the pulmonary endothelium and vascular smooth muscle. This has accompanied significant advances in the mechanistic and pathogenic understanding of PAH, which has led to the development of novel therapeutics [10], and has contributed to a significant improvement in outcomes for PAH patients over the past 3 decades [11]. Despite recent developments, however, PAH remains a disease with significant delays in diagnosis and profound morbidity related to systemic disease effects, in part related to the limitations of classic diagnostic approaches and due to the fact that current treatments largely do not reverse pulmonary vascular remodeling, respectively. Ultimately, PAH remains incurable and median survival is merely 7 years [12], a prognosis that is worse in various PAH subtypes, including systemic sclerosis (SSc)-associated PAH and in bone morphogenetic protein receptor type II (*BMPR2*) mutation carriers [13].

In this context, it follows that in some cases, resting supine hemodynamic measurements may be inadequate to optimally characterize the interactions within the right heart circulatory system and may insufficiently distinguish early pulmonary vascular dysfunction or disease. Similarly, while more dynamic approaches to characterizing interactions between the right heart and the pulmonary vasculature, including non-invasive cardiopulmonary exercise testing (niCPET), may be helpful for screening, risk assessment, and evaluation of therapeutic response, they lack measurement of invasive parameters that detect more subtle hemodynamic findings in PAH. As such, information from invasive cardiopulmonary exercise testing (iCPET) presents an important opportunity to enhance early diagnosis, phenotype disease subtypes, and personalize treatment in PAH given the shortcomings of these contemporary diagnostic and therapeutic approaches. By allowing for the simultaneous synthesis of invasive intracardiac and pulmonary pressures, minute-by-minute gas exchange and ventilatory parameters, and blood gas measurements, iCPET is able to evaluate the dynamic interactions within and between the right heart circulatory system and the skeletal muscle [14] during different loading conditions. This is central to improving our understanding of the hemodynamic, metabolic, and molecular underpinnings of PAH to improve functional and prognostic outcomes for patients. The purpose of this narrative review is to provide a framework for understanding the basis of aberrant exercise physiology in PAH and to discuss the clinical utility of iCPET in patients with PAH, with a special focus on two relevant and often overlooked pathophysiologic states in this population—exercise PH (exPH) and impaired systemic peripheral oxygen extraction (EO_2_).

## 2. Methods

The relevant literature was identified via the PubMed database (Bethesda, MD, USA), which is maintained by the National Center for Biotechnology Information at the U.S. National Library of Medicine. The database was searched for English-language articles up to 30 December 2024. Search terms related to PAH were utilized and included but were not limited to the following: “pulmonary arterial hypertension”, “non-invasive cardiopulmonary exercise test”, “invasive cardiopulmonary exercise test”, “advanced cardiopulmonary exercise test”, “exercise physiology”, “exercise pulmonary hypertension”, and “early pulmonary hypertension”. Abstracts were reviewed on PubMed and articles were accessed through the Yale Harvey Cushing/John Hay Whitney Medical Library and read in their entirety. Articles were accepted for inclusion based on relevance to clinical practice and contemporary research priorities in the PAH field; the overall quality of the selected articles was assessed by the full authorship team. When possible, randomized controlled clinical trials, large longitudinal observational cohort studies, meta-analyses, and manuscripts published within the last 10 years were included over smaller case series.

### 2.1. Exercise Physiology in Healthy Individuals

Under normal circumstances, performing exercise requires complex interactions between the cardiopulmonary system, the neurovascular system, and the components of cellular metabolism to ensure the efficient integration of the external and internal respiratory systems to balance energy production and energy consumption [15]. Muscular work is dependent upon an increase in oxygen consumption to generate adenosine triphosphate (ATP) from adenosine diphosphate (ADP) in the mitochondria, which is the primary source of cellular energy in muscle cells, and occurs via the processes of aerobic oxidation, anaerobic hydrolysis of phosphocreatine (PCr), and anaerobic catabolism [16]. The process linking these physiologic steps in the flow of oxygen from the mouth to the mitochondria to sustain exercise is known as the oxygen (O_2_) pathway, which maintains homeostasis in the setting of increasing work [17]. This physiologic cascade utilizes both convective and diffusive O_2_ delivery mechanisms and includes alveolar ventilation, oxygen diffusion into capillary blood, circulatory distribution of blood to exercising muscles through large blood vessels and microcirculation, O_2_ diffusion into cells/systemic peripheral EO_2_, and the utilization of oxygen in the mitochondria [15,17].

Based on the Fick principle, the O_2_ utilized throughout the O_2_ pathway is characterized as the oxygen uptake (VO_2_), which is also known as aerobic or exercise capacity, and distinguishes exercise limitations related to cardiopulmonary pathology [18]. VO_2_ is equal to the product of cardiac output (CO) and the arterial-mixed venous oxygen content difference (C(a-v)O_2_) and increases up to 20-fold during exercise [19]. Healthy adults have a maximum VO_2_ of 35–45 mL/kg/min, whereas patients with severe PAH may have a maximum VO_2_ closer to 15 mL/kg/min [20]. To maximize VO_2_ during exercise, CO increases via the initial augmentation of stroke volume, due to increased venous return from the venous capacitance vessels to the heart along with increased cardiac contractility [14,21]. The CO distribution through the macrocirculation is facilitated by the vasodilation of the contracting muscle along with the constriction of the splanchnic and renal vessels [22]. On the tissue level, the microcirculation adjusts blood flow and oxygen delivery (DO_2_) to maintain adequate tissue perfusion, in part through the increased production of vasodilatory substances including histamine, adenosine, and nitric oxide (NO) [23]. Ultimately, oxygen is extracted by the tissues, represented mathematically by the augmentation of C(a-v)O_2_ [19]. These processes are coupled to tightly regulated ventilatory changes including an increase in minute ventilation (Ve) and pulmonary gas exchange during exercise, and both must increase sufficiently to compensate for work-generated metabolic products, including carbon dioxide and lactic acid [16], the latter of which results from O_2_-independent metabolism at the onset of the anaerobic threshold (AT). Ultimately, at the point of maximal VO_2_ (VO_2_ max), which is identified as the O_2_ consumption plateau in the face of increasing intensity, physical activity will cease when the lactate concentration exceeds ventilatory capacity as well as endogenous buffering capabilities leading to metabolic acidemia [14].

The response of the right heart circulatory system—an integrated functional unit which comprises the systemic veins up to the pulmonary capillaries [24]—to the effects of an increased workload during exercise is unique given the distinct morphologic and physiologic components of the system [25], including the unique morphology of the RV, which has been described in detail previously [1,26,27,28]. During exercise, there is an increase in venous return to the right heart, facilitated by sympathetically mediated vasoconstriction, which moves blood toward the central circulation. Normal RV function is dependent on its preload, or the stretch of its wall at end-diastole prior to contraction; afterload, or the hydraulic load imposed by the pulmonary vasculature; native contractility and pericardial compliance [29]. Because the RV walls are thin and compliant with a low volume-to-wall surface area ratio, it is able to accommodate exercise-related increases in preload well [3]. However, this ability depends on the maximum capacity of its longitudinal circumferential oblique muscle fibers to shorten, and when it is exceeded, RV contraction suffers and SV decreases [30]. Conversely, the RV tolerates afterload poorly, as it is coupled at baseline to the highly compliant and low-resistance pulmonary circulation, and is highly sensitive to pressure-loaded conditions as explained by its shallow end-systolic pressure–volume slope [29]. Both steady and pulsatile components impact RV afterload, and the former related to the opposition to flow and the latter related to the energy required to overcome systolic pressure [31].

In healthy conditions, optimal ventriculoarterial coupling, also known as RV-PA coupling is dependent on maximally efficient energy transfer from the RV to the pulmonary circulation [32]. This process is described by the quotient of end-systolic elastance (*E_es_*) and effective arterial elastance (*E_a_*), which refers to afterload. RV-PA coupling is integral to ensuring that increased contractility does not lead to disproportionate increases in pulmonary pressures. In healthy conditions, the RV is able to increase *E_es_* in response to an increase in *E_a_* even during peak exercise and beyond the ventilatory threshold [33]. Its adaptive response includes homeometric adaptation, characterized by increased contractility, followed by concentric RV hypertrophy in the absence of dilation and fibrosis [28,32].

The lung must be able to accommodate an increase in volume and flow generated by the LV [14]. During exercise, this increase in flow, in addition to an increase in LA pressure and right atrial (RA) pressure, leads to an increase in pulmonary artery pressure (PAP) that is proportional to the rise in CO and is affected by workload and age [34,35]. Additionally, with exercise, there is a modest decrease in the PVR, which is attributed to the recruitment of small pulmonary resistive vessels followed by their distention to allow for increased red blood cell transit, which ultimately leads to a decreased pulmonary arterial compliance (PAC) related to pulmonary resistance (Rp) (which is represented by a decline in the Rp-PAC time product) [18,36,37]. Total pulmonary resistance (TPR) is also observed to decrease during exercise in young patients, which is accounted for by a decrease in PVR in addition to left ventricular filling resistance; for older patients, there is often a delay in TPR decrease during exercise, which may be due to delayed reduction in left ventricular filling resistance due to latent left heart dysfunction [38].

### 2.2. Abnormal Exercise Physiology in PAH as Described by Non-Invasive Cardiopulmonary Exercise Testing in PAH

A basic understanding of aberrant exercise physiology in PAH can be achieved through non-invasive testing. This includes non-invasive CPET (niCPET), which is an important tool in PAH and has its own robust body of literature that has been described elsewhere in detail [39,40,41,42,43,44]. niCPET allows for an analysis of cardiovascular variables during exercise that facilitates the identification of abnormal hemodynamics but cannot reliably distinguish patterns of exercise among patients with PAH, heart failure, and PH related to left heart disease [18]. It is the gold-standard approach to evaluating aerobic capacity via VO_2_ measurement in addition to HR and ventilation at maximal exercise levels [45], and it can rule out a pulmonary mechanical limit to exercise as defined by a low breathing reserve (the difference between the maximal voluntary ventilation and the maximum exercise ventilation), which is an uncommon primary deficit in patients with PAH [16,46]. However, while niCPET can suggest central cardiovascular dysfunction—including heart failure or pulmonary vascular disease (PVD)—or impaired peripheral EO_2_ as causes of low VO_2_, it cannot reliably distinguish between these entities since it does not provide direct measurements of CO or cardiopulmonary hemodynamics, which is critical to understand disease pathophysiology [14].

With that said, in PAH, there are several niCPET cardiovascular parameters that can help to assess functional class and disease severity; these include peak VO_2_, VO_2_ to work rate ratio, HR, O_2_ pulse (VO_2_/HR), and O_2_ uptake efficiency slope (OUES), the latter of which is particularly useful to assess prognosis in PAH during submaximum exercise testing [20,44,47]. In particular, low VO_2_, which reflects an impaired ability to increase pulmonary blood flow during exercise, has been shown to aid in diagnosis, functional assessment, and prognosis in PAH [41], including in those with SSc. [48]. VO_2_ values are thus utilized in clinical risk stratification tools [4]. For instance, peak VO_2_ is an independent predictor of mortality in patients with PAH, as well as a predictor of time to clinical worsening [49,50,51]. Changes in peak VO_2_ can also be used to track responses to PAH therapy [52,53] and have been negatively associated with dynamic PAC during exercise as well as with load dependent measures of RV contractility including the RV stroke work index [54]. Additionally, VO_2_ has been shown to be both a sensitive and specific discriminator between SSc patients with and without PAH [55]. In addition to low VO_2_, niCPET can be useful in characterizing another measure of aerobic fitness in PAH, the AT, which reflects the highest sustained VO_2_ prior to the development of lactic acidosis [16]. In patients with PAH, exercise causes a disproportionate increase in RV pressure and RV dilation, prompting interventricular septal distortion and decreased LV preload, SV, and ultimately CO. This leads to the early generation of lactic acidosis due to inadequate tissue DO_2_ in combination with arterial desaturation, and thus a reduced AT, which has been shown to be an independent marker of PAH disease severity [42,47]. Newer pathophysiologic data using iCPET have further indirectly characterized the impact of impaired oxygen utilization at the muscle level contributing to early lactic acidosis in this patient population, which is described in detail in later sections.

Patients with PAH are known to have other typical patterns on niCPET including oxygen desaturation, as a result of low mixed venous oxygen saturation (MVO_2_), high physiologic dead space, and/or possible right-to-left shunts (e.g., in the case of a patent foramen ovale); a high ventilatory equivalent for carbon dioxide (VE/VCO_2_); and a low end-tidal partial pressure of carbon dioxide (P_ET_CO_2_) [14,44,56]. These values can help support a diagnosis of PAH, assess disease severity, and gauge therapeutic responses to PAH medications [43]. For instance, the Ve/VCO_2_ slope is higher in patients with PAH due to the impaired distention and recruitment of pulmonary vessels, leading to worsening ventilation–perfusion matching and an increased dead space to tidal volume ratio. This marker of ventilatory inefficiency is a classic finding in PAH, and has been shown to predict survival not only in this population but also in patients with SSc without PAH, in whom developing pulmonary vasculopathy is a concern [18,48]; like VO_2_, ventilatory efficiency values are included in guideline-directed risk stratification approaches [4]. These effects are compounded by hyperventilation due to increased physiologic dead space and increased chemosensitivity, the latter of which is incompletely understood, but posited to be related to impaired RV lusitropy and increased sympathetic activation [57,58]. This pathophysiology is accompanied by an increased reliance on anaerobic metabolism in PAH, which drives CO_2_ production, leading to an abnormal ventilatory response reflected in a reduced P_ET_CO_2_ both at rest and with exercise in PAH [59]. In fact, depressed P_ET_CO_2_ has been shown to correlate with disease severity in PAH and raises strong suspicions for the diagnosis of PAH, especially in the absence of acute hyperventilation and in the setting of arterial hypoxemia [59].

### 2.3. Invasive Cardiopulmonary Exercise Testing in PAH

#### 2.3.1. Clinical Considerations

iCPET expands the scope of data extracted from niCPET for patients with PAH. It allows for the collection of complete cardiopulmonary hemodynamics and peripheral tissue EO_2_ analyses to assess the interactions between the RV, pulmonary circulation, and skeletal muscle [60]. These data facilitate a more nuanced approach to understanding the pathophysiology of PAH, allow for the critical diagnosis of early or exPH; are a tool to assess response to PAH therapy, and help to characterize individual functional limitations in patients with established PAH, which include diverse etiologies such as concomitant exercise-induced heart failure with preserved left ventricular ejection fraction (HFpEF), preload-dependent limitations to cardiac output (“preload failure”), and impaired peripheral systemic EO_2_, which is variably referred to in the literature by other terms including neurovascular dysregulation [46,61].

In patients who present with exercise limitations or dyspnea, including those with previously diagnosed PAH, it is primarily important to perform thorough non-invasive testing, which includes, but is not limited to a physical exam, serologic studies, cross-sectional chest imaging, electrocardiogram (ECG), transthoracic echocardiogram (TTE), 6 min walk test/ambulatory oximetry, and pulmonary function testing (PFT). This may be followed by more comprehensive cardiopulmonary evaluations including resting supine RHC or left heart catheterization, and niCPET. These aforementioned diagnostic modalities can facilitate the diagnosis of common cardiopulmonary conditions in patients with PAH including interstitial lung disease (ILD), chronic obstructive lung disease (COPD), high-output heart failure (HOHF), secondary to pulmonary vasodilator treatment, and various cardiomyopathies (including left ventricular systolic dysfunction or HFpEF), in addition to demonstrating the progression of PAH. In patients with PAH with ongoing exercise intolerance, who have an otherwise unrevealing non-invasive evaluation, iCPET should therefore be considered to clarify the pathophysiologic underpinnings of dyspnea to inform therapeutic intervention. iCPET should similarly be considered in patients without PAH who have dyspnea of unknown etiology with an unrevealing non-invasive evaluation. This point is particularly relevant for patients with risk factors for PAH, including those with connective tissue disease (e.g., systemic sclerosis) or first-degree relatives with PAH, in whom there is suspicion for exPH.

#### 2.3.2. Technical Considerations

**Figure 1:**
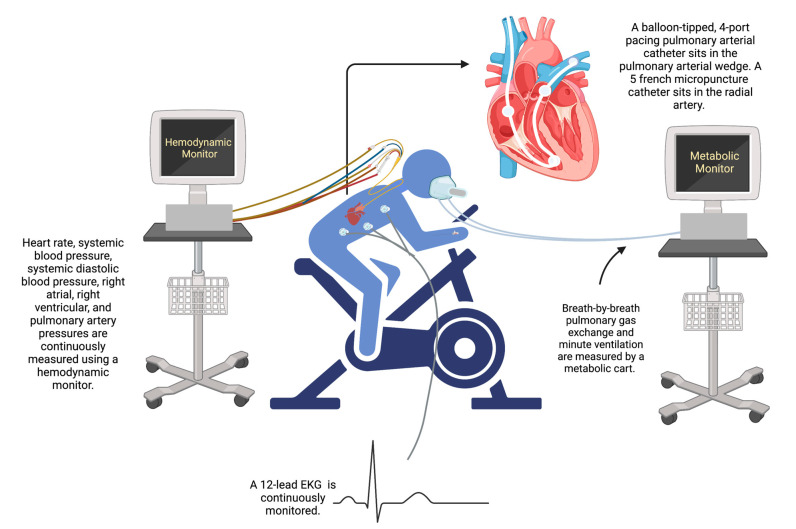
The invasive cardiopulmonary exercise test.

iCPET is a safe and well-tolerated procedure that involves interdisciplinary collaboration between pulmonologists, cardiologists, respiratory therapists, and exercise physiologists [14]. This procedure is felt to have similar risks to that of resting RHC provided patients do not have unstable disease (i.e., in the setting of active arrhythmias) or decompensated right heart failure [62,63]. Contraindications to the procedure are similar to those of non-invasive cardiac stress testing and niCPET [64,65], with additional considerations related to the safety and feasibility of PA catheter and radial artery catheter placement including hematologic status, venous accessibility issues, anticoagulation status, and the presence of underlying vasculopathy [14,66]. iCPET involves the deployment of a pulmonary artery catheter in the internal jugular vein under fluoroscopic guidance to avoid expected difficulties with exercise related to a brachial or femoral approach [44]. A standard resting supine RHC is completed as recommended by current guidelines, and the catheter is adjusted in the ideal position to allow for the collection of PAWP during exercise and is secured in place [4]. In our lab, we currently use a fluid-filled PA catheter to record hemodynamic data at rest and during exercise, but these may be subject to artifact, poor fidelity, and pressure dampening [60], and thus a more optimal though less accessible option is the use of a micromanometer-tipped catheter, which provides a higher frequency response with higher quality pressure waveforms [46]. We then recommend placing a radial artery catheter under ultrasound guidance for the collection of arterial blood gasses (ABG) during exercise to directly measure arterial oxygen pressure (P_a_O_2_) to calculate the alveolar–arterial difference for oxygen pressure [P(a–a)O_2_] [64]. An arterial catheter also allows for the collection of oxygen saturation (S_a_O_2_) to ensure accurate Fick CO calculation [67], the importance of which was noted in a recent report by Campedelli et al., which highlighted poor correlation between S_a_O_2_ and peripheral arterial oxygen saturation (S_p_O_2_) during iCPET, leading to the misclassification of patients with both pre- and post-capillary ePH [67], which may lead to incorrect therapeutic interventions. After catheter placement, the patient is brought to the exercise lab for CPET (Figure 1).

In our exercise lab, similar to others, patients perform a maximal exercise test (performed until peak individual exercise) on an upright cycle ergometer with a mouthpiece that allows for the collection of gas exchange parameters [68,69]. The cycle ergometer is chosen over a treadmill exercise protocol in an effort to minimize motion artifact during the collection of intracardiac pressures and to facilitate a continuous ramp protocol, and because it is the primary method used in clinical trials [44]. The upright position, while perhaps more susceptible to “whip” artifact and PAWP variability due to west zone measurement inconsistency [46], is felt to offer several benefits over supine exercise in PAH patients including greater clinical relevance, more physiologic positioning, and the patient’s ability to achieve a high work rate during exercise [70], allowing for a more robust evaluation of the hemodynamic response to exercise during an activity that mimics daily activities and avoids a supine-related peripheral sympathetic mechanoreflex [71]. With that said, upright positioning in PAH patients may lead to higher PVR at rest and end-exercise, and a higher end-exercise mPAP and mPAP/CO slope [70]. As such, regardless of position, clinicians should carefully weigh the consequences of the choice during the interpretation of data to avoid unintended confounding variables.

The zero reference level for iCPET is set at the level of the LA, or the horizontal level of the phlebostatic axis, which corresponds to the point of intersection between the frontal plane at mid-thoracic level, the transverse plane at the level of the fourth intercostal space, and the midsagittal plane [68,72]. Each minute during exercise, direct Fick CO, which corresponds to total pulmonary blood flow (as long as there are neither shunts nor bronchial-to-pulmonary artery collateral blood flow) is calculated using direct measurements of VO_2_ and ABG and mixed venous blood gas samples, though it is also reasonable to determine CO by thermodilution, recognizing that this may be difficult due to the conditions of exercise at high workloads [63]. Blood gas data are also used to determine acid–base status, calculate P(a–a)O_2_, calculate the dead space fraction, and analyze peripheral EO_2_ variables [44]. Additionally, mean systemic arterial pressure, RA pressure, systolic/diastolic and mean RV pressure, and systolic/diastolic and PAP are recorded continuously, and PAWP is recorded each minute until peak exercise is reached [14].

With these data, TPR (mPAP/CO), PVR (mPAP-PAWP/CO), mPAP/CO, and PAWP/CO slopes are calculated. Additionally, with 4–5 measurements at increasing workloads, it is possible to calculate the distensibility coefficient α, which refers to the percentage change in diameter of pulmonary resistive vessels per change in distending pressure [44,68,73]. Here, it is important to consider the impact of intrathoracic pressure—reliant on alveolar pressure, lung volume, ventilatory flow, pulmonary elastance, and airway resistance—on pulmonary vascular pressures, which is greater with increased ventilation during exercise in normal subjects and accentuated in patients with dynamic hyperinflation related to obstructive airway disease [74]. While historical data suggested the measurement of end-expiratory pressure to avoid the aforementioned wide pleural pressure variations, more recent studies support averaging mPAP and PAWP over the respiratory cycle, especially in patients with chronic obstructive pulmonary disease (COPD) and obesity [63,72,74].

#### 2.3.3. Special Considerations

While performing iCPET remains the gold-standard approach to understanding the pathophysiology of dyspnea and exercise intolerance in patients both with and without PAH, and in most cases can inform clinicians regarding response to treatment and prognosis in patients with PAH, there are various technical concerns related to the practical application of this testing. For instance, there is no standard approach to conducting iCPET [60]. As described above, several distinct protocols exist for various portions of the exam including upright versus supine cycling [70], the use of direct Fick CO measurement versus thermodilution CO measurement, the use of a fluid-filled PA catheter versus a micromanometer-tipped PA catheter [63], and whether or not serial ABGs are collected to determine S_a_O_2_. Data from iCPET must thus be interpreted with attention to its distinct procedural approaches, which often requires a clinician who is comfortable with the nuances of this testing modality. iCPET also remains underutilized despite its utility [14]. For instance, patients may be uncomfortable with the invasive nature of the testing, though this can usually be overcome with personalized counseling and education by the performing physician. Additionally, there is a mismatch between the demand for and supply of iCPET, largely due to difficulties around expedient referrals [75] related to the fact that iCPET is typically only performed at academic medical centers given its requirement of robust supporting infrastructure [69]. For instance, the minimum staffing required to maintain an iCPET program includes a supervising physician (which can be a cardiologist or pulmonologist), at least 1 catheterization lab nurse and catheterization laboratory technician, a respiratory therapist, a scheduler, and an exercise physiologist for the interpretation of results, all of which may be difficult to support based on the institution [69]. Finally, in addition to requiring specialized equipment, well-trained providers, and dedicated facilities, there are high operational costs of maintaining the program with low relative value units (RVU) for the amount of work performed, and thus programs rely heavily on institutional support and grant funding [69]. With that said, the overall holistic value of iCPET for patients is significant, and its performance at expert centers has been shown to reduce the time to diagnosis compared with traditional treatment and testing, and can potentially reduce the number of diagnostic tests and unnecessary treatments patients experience [75].

### 2.4. Exercise Pulmonary Hypertension: A New Definition Using iCPET Parameters

#### 2.4.1. History

The European Society of Cardiology/European Respiratory Society (ESC/ERS) reintroduced exPH in the 2022 PH guidelines, defined by an mPAP/CO slope > 3 mmHg/min between rest and exercise [4,76], which was upheld at the Seventh World Symposium on Pulmonary Hypertension in 2024 [68]. Of note, while exPH is a condition that is hemodynamically defined by parameters obtained during iCPET and may represent a form of early PH, it is a distinct concept from early PH, which often refers to patients with a resting PAP between 20 and 25 mmHg and a PVR of 2–3 WU. The decision to include a definition of exPH in the guidelines followed a long evolution in the scholarship of exercise-related hemodynamics, which have been studied since the development of the Swan–Ganz or PA catheter and its practical application during the RHC [60,76]. Historical scholarship led to the original consensus that mPAP should not exceed 30 mmHg at peak physical activity, thus supporting an early definition of exPH as mPAP > 30 mmHg during exercise, without the integration of other hemodynamic parameters. This was discussed at the first WHO meeting on primary PH in 1973 and was included in the 2004 ESC guidelines on PH, leading to practical adoption by clinicians [35,77]. However, there were ongoing questions around whether an mPAP > 30 mmHg during exercise was necessarily pathological, in part driven by the observation that highly trained athletes consistently exceed an exercise-related increase in mPAP > 30 mmHg largely due to increases in SV [63].

#### 2.4.2. Supporting a Definition Based on the mPAP/CO Slope

An initial answer to the question of what constitutes abnormal pulmonary hemodynamics during exercise was provided by a large meta-analysis of iCPETs (that included heterogeneous exercise methods) during rest and exercise in healthy individuals, which demonstrated that it is impossible to define a single upper limit of normal mPAP that holds for all individuals at all exercise levels. This study revealed that exercise mPAP is age related and found that up to 21% of subjects aged <50 years and 47% of subjects >50 years surpassed the previously defined threshold for exPH during maximal exercise, though in the younger patients this was associated with high CO [34]. These findings contributed to the decision to abandon the definition of exPH as mPAP >30 mmHg at the Fourth World Symposium on Pulmonary Hypertension and to not include it in the ESC/ERS PH guidelines in 2009 and 2015 [78,79]. This was followed by robust analyses of normal exercise hemodynamics and of the prognostic relevance of exercise hemodynamics, including in a large systematic review, which revealed that mPAP is linearly (or almost linearly) related to CO in individuals under 50, with a steeper slope in older individuals likely due to the age-related loss of PAC and increased LV diastolic stiffness, supporting the notion that abnormalities in exercise mPAP cannot be defined by a single value and instead must be defined relative to CO, through the mPAP/CO slope [38]. A subsequent study of subjects with preserved exercise capacity used iCPET with upright cycle ergometry clarified the age-related upper limits of normal (ULN) for various iCPET variables including for the mPAP, PAWP, and mPAP/CO slope [80]. Finally, in a second systematic review and meta-analysis, it was clarified that the mPAP/CO slope during exercise is a reliable variable to characterize a pulmonary vascular response to exercise, finding it is always positive and related to age. In this analysis, the weighted mean of the slope ranged from 1.6 to 3.3 mmHg/L/min in subjects aged 30–70 years old. Specifically, an mPAP/CO slope >3 mmHg/L/min was not observed in healthy subjects less than 60 years old, and only rarely in subjects >60 years old, identifying this cutoff as a marker of pathophysiology [76], thus leading to this value’s adoption in the current guidelines as the hemodynamic definition of exPH. With that said, it is important to note that these aforementioned data supporting the current guidelines have largely been based on the ULN in healthy individuals, which may have overrepresented fit individuals and retrospective single-center data reporting the association between mPAP/CO slope >3 and cardiopulmonary outcomes, and thus lacks the robust quality of large, multicenter studies [81].

#### 2.4.3. Distinguishing Between Pre-Capillary and Post-Capillary Causes of exPH

The mPAP/CO slope may be elevated in the setting of pulmonary vascular remodeling or in the setting of left heart disease with elevated left-sided filling pressures, and thus does not differentiate between pre- and post-capillary causes of exPH [82]. Multiple studies have thus explored exercise parameters to distinguish right and left heart disease based on exercise parameters. A study by Eisman et al. showed that PAWP increases linearly to CO and that a cutoff of PAWP/CO >2 mmHg/L/min most accurately suggests left heart pathophysiology with steeper slopes associated with worse aerobic fitness and adverse clinical outcomes, even in the absence of abnormal resting PAP and PAWP or a HFpEF diagnosis [83]. The association of prognosis with an elevated PAWP/CO slope was similarly elucidated by Ho et al. [84]. The aforementioned findings were supported by data showing that among healthy patients, the PAWP/CO slope is almost always ≤2 mmHg/L/min [37]. Ultimately, the systemic review and meta-analysis by Zeder et al. again suggested that available data support a PAWP/CO > 2 mmHg/L/min as the most prognostically relevant cutoff for exPH due to left heart disease, likely because it incorporates the variable of exercise-related changes in blood flow [76]. Due to these findings, current guidelines suggest that a PAWP/CO > 2 mmHg/L/min between rest and exercise suggests a post-capillary cause of exPH [4]. Of note, at the most recent World Symposium of Pulmonary Hypertension, it was suggested that an increase in the absolute value of peak PAWP >25 mmHg was also reflective of post-capillary exPH [68], echoing the ESC recommendations for diagnosing HFpEF, which use an exercise PAWP cutoff of >20–23 mmHg [21]. This reflects multiple studies that have identified left heart disease as the primary cause of exercise impairment using absolute PAWP cutoffs of 20 mmHg or 25 mmHg [85,86,87], and work that has demonstrated increased mortality in HFpEF patients who experience a steep increase in PAWP during exercise to > 25 mmHg, though seminal studies describing this used supine exercise, which is not the current standard [87].

**Figure 2:**
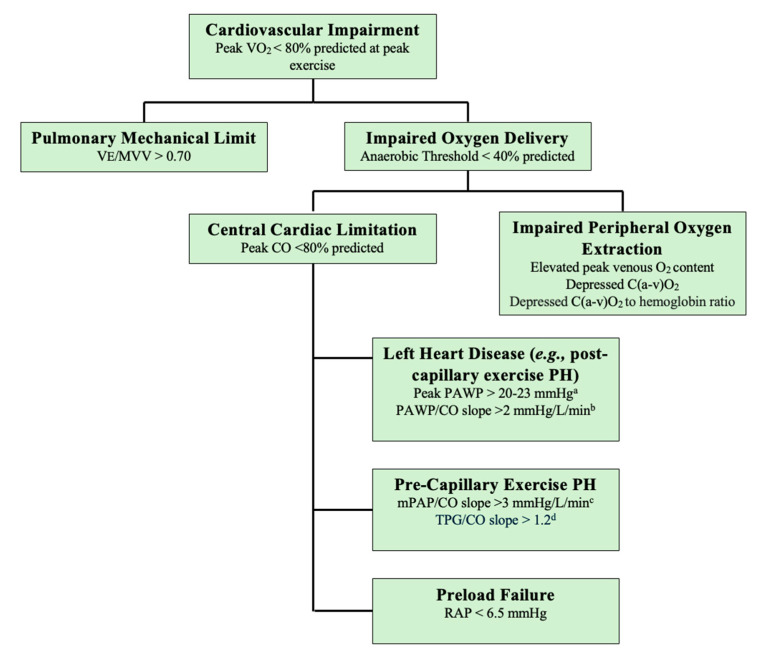
The iCPET can differentiate central cardiac and peripheral limitations to acute exercise (once eliminating the existence of pulmonary mechanical limitations to exercise). Central cardiac limitations can be due to left-sided heart disease (e.g., HFpEF), right-sided heart disease (e.g., pre-capillary exPH), or inadequate cardiac preload. ExPH can be further delineated into pre-capillary and post-capillary etiologies. Impaired systemic oxygen extraction, which is also known as a peripheral limitation, may be due to mitochondrial myopathy or microcirculatory left-to-right shunts. ^a,b^ The PAWP/CO slope is age dependent. ^c,d^ The mPAP/CO slope is age dependent, whereas the TPG/CO slope is age independent. Exercise pulmonary hypertension: prognostic relevance in PAH.

Additional hemodynamic measures to discern pre-capillary exPH are more conceptual and not included in the current guidelines, though abnormalities in peak PVR, TPR, TPG, and the TPG/CO slope can suggest PVD [76]. Briefly, Oliveira et al. showed that there are significant age-related differences in the pulmonary vascular response to exercise in normal subjects with peak exercise PVR ULNs of 2.1 WU for subjects > 50 years old and 1.34 WU for subjects ≤50 years old [80]; however, a higher-than-expected PVR in an older individual may reflect the inability to decrease left ventricular filling resistance with increased CO and thus may not indicate isolated pre-capillary disease. Additionally, TPR should not exceed 3 WU during exercise [73,82] and has been shown to discriminate between PVD and left heart disease in patients with mPAP > 30 mmHg [88]. Of note, the TPG–flow relationship has been shown to be linear and age-independent, with normal TPG/CO slope values of 0.8 ± 0.2 mmHg/L/min (ULN 1.2 mmHg/L/min) [76]. This value may have particular utility because it is not affected by intrathoracic pressure swings since mPAP and PAWP are both equally affected [63] (Figure 2 [89]).

There is growing evidence suggesting that an abnormal pulmonary vascular response to exercise is clinically relevant with significant prognostic value (Table 1). In fact, exPH is increasingly being recognized as a precursor to PAH, or early PH [90], and is commonly diagnosed in patients with mild (“borderline”) PH, pointing to a probable association between these conditions [91].

In a formative retrospective study of 700 individuals with chronic exertional dyspnea, Ho et al. found that clinical determinants of exPH, including the mPAP/CO slope, the TPG/CO slope, and the PAWP/CO slope are associated with worse functional capacity and abnormal right ventricular contractile reserve; additionally, the presence of exPH was shown to predict worse cardiovascular event-free survival [84]. Importantly, the poorer prognostic indicators of exPH were consistent even in individuals without resting PH, supporting the pursuit of iCPET to clarify exercise hemodynamics in patients with unexplained dyspnea [84]. Similarly, data from Douschan et al. confirmed the prognostic relevance of exPH in over 200 patients with normal or mildly elevated mPAP <25 mmHg [92]. This group showed that independent of age, sex, smoking status, functional class, and cardiopulmonary comorbidities, the mPAP/CO slope, PAWP/CO slope, TPG/CO slope, and peak CO were independent predictors of mortality; additionally, like Ho et al., this group confirmed the association between mortality and worse exercise hemodynamics in the absence of abnormal resting hemodynamics [84]. This is supported by earlier studies showing that exercise-related increases in mPAP and PVR are associated with decreased exercise capacity and measurements of cardiac function [90], and that in a general population, patients with an mPAP of 18–20 mmHg have a poorer prognosis compared to those with lower resting pressures [93]. Additionally, in a large cohort of patients with a normal resting mPAP and PAWP (and without a diagnosis of HFpEF), Eisman et al. found that an abnormally steep PAWP/CO slope during exercise is associated with poor aerobic fitness and adverse clinical outcomes, again supporting the ability of exercise to uncover more subtle disease phenotypes, which is relevant both for surveillance purposes and for possible early application of therapy [83].

Most recently, in perhaps the most robust study to date, the Pulmonary Haemodynamics during Exercise Network Clinical Research Collaboration (PEX-NET CRC) published the results of the first large, international multi-center retrospective study of the prognostic relevance of exercise pulmonary hemodynamics in patients presenting with a resting mPAP < 25 mmHg. In this study of over 700 patients using heterogeneous exercise protocols, an mPAP/CO slope > 3 mmHg/L/min was shown to be a robust, independent predictor of mortality (mortality hazard ratio 2.04) [81]. These findings support both the current definition of exPH as mPAP/CO slope > 3 mmHg/L/min and point to its use as a prognosticator in patients with only minimally abnormal hemodynamics.

The prognostic value of exercise hemodynamics especially appears to be the case in populations at risk for developing PAH. For instance, in asymptomatic *BMPR2* mutation carriers, who have an annual risk of incident PAH of 2.3% [94], Gerges et al. found that the presence of exPH as defined by mPAP/CO > 3 mmHg/L/min, had a 81% specificity and 60% sensitivity for developing PAH during long-term follow-up [95]. Perhaps more interesting, this group found that the earlier identification of *BMPR2* mutation carriers at risk for PAH was possible with the iCPET assessment of the distensibility coefficient α. Not only was an α ≤ 1.5%/mm able to predict PAH occurrence with a specificity of 75% and sensitivity of 100% in this population, but patients who went on to develop PAH had a lower α at baseline [95]. The latter point suggests occult mild pulmonary vascular remodeling may exist in this patient population, not appreciable with non-invasive testing. Additionally, in SSc patients, a population with an increased risk of developing PAH [96], exPH has been posited to represent an early form of pulmonary vasculopathy. Early studies [97], including by Kovacs et al., found that in patients with SSc, an mPAP and PVR in the upper normal range at rest is associated with decreased exercise capacity [98]. The Kovacs group also used exercise echocardiography and RHC to assess SSc patients over the course of four years and found that over this time period there was a significant increase in the PAP, PVR, and mPAP/CO slopes during exercise, along with a significant decrease in functional capacity that did not translate into a significant increase in resting values [99]. More recently, using combined pressure and flow-related criteria (mPAP > 30 mmHg and TPR > 3 WU during exercise) to define exPH in an SSc population with normal resting hemodynamics, Stamm et al. found that patients with exPH had a reduced transplant-free survival that was similar to SSc patients with PH at rest [100]. Importantly, the exPH group in this study had a slightly higher mPAP and PVR at rest compared to patients without PH. In a more recent study investigating pulmonary vascular resistances during exercise in SSc patients, Zeder et al. found that peak exercise PVR, TPR, mPAP/CO slopes, and the TPG/CO slope have prognostic relevance [101]. Specifically, in a cohort of patients that had a resting mPAP <25 mmHg, survival was associated with the PVR, CO, and mPAP/CO slope at peak exercise, and an mPAP/CO slope of <3.5 WU was associated with 100% 10-year survival [101]. Together, these studies suggests that in addition to being prognostically relevant, exPH may be an early phase of PVD, or may predict the future development of PAH.

The assessment of PAC and pulmonary vascular distensibility has aided explorations into whether exPH represents an early form of PVD. It has been shown that patients with exPH or mild PH have reduced PAC, which suggests that PAC decreases prior to the overt appearance of PAH and may support the notion that vessel stiffness contributes to the progression of disease by increasing pulse pressure and augmenting PA dilation, thus accelerating the effects of shear stress and distal vasculopathy [31,102]. Distensibility has also been shown to be reduced in patients with mild PVD without an overt resting PH (even in patients with mPAP ≤ 20 mmHg), indicating that dysregulation in this property is a relatively early finding in this disease process [103]. This was explored by Wallace et al. who demonstrated that patients with exPH and normal resting hemodynamics, including those with SSc, have physiologic evidence of early PVD, including a reduced PAC at rest and peak exercise and a reduced distensibility coefficient α, suggesting the existence of subclinical endothelial dysfunction, perhaps due to the early interruption of pulmonary arterial elastic lamina early in the disease process [31,104]. The role of abnormal pulmonary vascular distensibility as a sensitive marker of early PVD in patients with exPH was further investigated by Singh et al. who showed that decreased PV distensibility was associated with decreased aerobic capacity and dynamic RV-PA uncoupling, again suggesting early resistive vessel pathology similar to PAH [36]. Altogether, these data support a potential role of iCPET to unmask early PVD in patients at risk for developing PAH, a pathophysiologic process which may not be readily apparent with resting data analysis.

### 2.5. Exercise Pulmonary Hypertension: Treatment in Patients at Risk for PAH

Diagnosing exPH facilitates the detection of early PVD in patients at risk for developing overt abnormal resting hemodynamics and an understanding of the etiology of exertional dyspnea. However, treatment is less well established compared to resting PH, regardless of cause, and neither current guidelines nor consensus statements provide clear treatment recommendations for exPH [4,105]. However, given that the pathobiology of pre-capillary exPH likely involves endothelial dysfunction, perhaps related to reduced endogenous NO production during exercise [106], there is a reasonable conceptual basis for the therapeutic targeting of dysfunctional endothelium with traditional pulmonary vasodilators [104]. The practical rationale supporting the treatment of exPH with traditional pulmonary vasodilator therapy is extrapolated from data from a patient population with SSc and in a population with mildly elevated resting hemodynamics, and with variable definitions of exPH; thus, generalizability has not been established.

Multiple small studies have tested the use of ambrisentan in patients with exPH. An open-label study was performed in patients with SSc and an mPAP > 30 mmHg and TPG > 15 mmHg during maximal exercise and found that treatment improved patients’ exercise hemodynamics (PVR) and exercise capacity [107]. These findings were supported by a small randomized placebo-controlled trial that evaluated the effect of ambrisentan on patients with SSc with a pulmonary vascular phenotype without ILD or left heart disease (resting mPAP between 21 and 24 mmHg and PAWP ≤ 15 mmHg and/or exercise mPAP > 30 mmHg and PAWP <18 mmHg) during submaximal exercise and found significant improvements in peak TPR, CO, and CI in the treated population [108]. During a mean follow-up time of 2.59 years, patients treated with ambrisentan showed improvement in resting RHC parameters and were less likely to develop overt PAH (as defined by an mPAP > 20 mmHg and PVR > 2 WU) [109], suggesting that treating exPH as an early form of PAH may attenuate the progression of disease. In another study, the treatment of participants with exPH defined by peak exercise mPAP > 30 mmHg, PAWP < 20 mmHg, and PVR > 1 WU led to significant improvement in exercise pulmonary hemodynamics including PAC, symptoms, and functional status, though with only a small trend toward improvement in VO_2_ [110]. Another small pilot study explored the use of bosentan in patients with SSc and a resting mPAP <25 mmHg (and PAWP ≤ 15 mmHg) and >30 mmHg during exercise and found that the progression of resting and exercise hemodynamics toward overt PAH were attenuated with treatment [111]. More recently, patients with exPH and lung disease and SSc as defined by peak exercise mPAP  ≥ 30 mmHg and TPR  ≥  3 WU or an mPAP/CO slope >  3.0 WU who were treated with various pulmonary vasodilators—including sildenafil, tadalafil, riociguat, and combination therapy with ambrisentan and tadalafil—were shown to have significant improvement in exercise hemodynamics including PAC; additionally, this has been the only study to date to show that treatment with exPH improves the distensibility coefficient α, which is posited to be a sensitive marker for early disease detection [103,104]. With that said, further research is required to validate the benefit of treating exPH in patients at risk for PAH with traditional pulmonary vasodilator therapy. Additionally, though beyond the scope of this review, there is a need to expand research into therapeutic options for patients with post-capillary exPH, building on promising new studies exploring the use of therapies such as sodium-glucose cotransporter-2 (SGLT2) inhibitors in this patient population [112,113].

Given both the prognostic relevance of exPH and the fact that it likely represents early PVD in distinct patient populations, in our practice, we consider offering treatment to carefully selected patients who meet the following criteria: 1) they have demonstrated exPH with an mPAP/CO slope > 3 WU with a pulmonary vascular disease phenotype suggested by a peak exercise PVR above the ULN based on age along with evidence of the abnormal widening of the alveolar–arterial (A-a) gradient and a failure of the expected decrease in dead space ventilation during exercise; 2) they have no evidence of left heart disease including a peak exercise PAWP/CO slope < 2 WU, resting PAWP <15 mmHg, and preferably few or no risk factors for left-sided cardiac disease; 3) they have risk factors for progression to overt PAH (e.g., connective tissue disease or known genetic mutations); and 4) they have a high symptom burden suspected secondary to exPH with other more common causes of cardiopulmonary disease excluded with non-invasive testing. In these patients, we will consider treatment with a single pulmonary vasodilator—either a phosphodiesterase 5 inhibitor or endothelin receptor antagonist—with patient-centered counseling regarding the off-label use of said therapies and very close follow-up for the management of adverse medication effects. Given the risk of disease progression, we regularly screen these patients with a multimodal strategy that includes regular clinical exams, serologic markers (e.g., NT-proBNP), transthoracic echocardiogram, and sub-maximal niCPET [68].

### 2.6. Beyond exPH: The Use of iCEPT to Phenotype PAH-Related Exercise Impairment

Patients with PAH may present with dyspnea and exercise intolerance despite the optimization of PAH-directed therapy, which may be due to dynamic interrelated mechanisms including the potential progression of PVD; other mechanisms involve impaired ventilation, respiratory muscle abnormalities, dysregulated cerebrovascular function, comorbid systemic diseases including renal disease and hematologic abnormalities, impaired cardiac function (including both RV and LV dysfunction), and skeletal muscle microcirculatory pathology (i.e., impaired peripheral systemic *EO_2_*) [30]. In these patients, it is critical to perform thorough non-invasive testing including a physical exam, electrocardiogram, transthoracic echocardiography (TTE), ambulatory oximetry, pulmonary function testing, and, if indicated, niCPET, resting supine RHC, left heart catheterization (LHC), and cardiac MRI, which can help to diagnosis common conditions such as iron deficiency anemia, lung disease, and left or right heart failure [114]. In the absence of a clear diagnosis, especially in light of near-normalized resting pulmonary hemodynamics, iCPET should be considered to elucidate symptom pathophysiology; this mode of testing has particular value in clarifying complex cardiopulmonary circulatory hemodynamics and in diagnosing impaired peripheral *EO_2_* in patients with PAH, the latter of which will be discussed in the remainder of this review.

### 2.7. Impaired Peripheral EO_2_ in PAH: Pathophysiology

While the major central cardiopulmonary abnormality in PAH is related to the negative impact of increased RV afterload on stroke volume augmentation and thus CO, there is growing recognition that this is not the entire story. More recently, it has been suggested that aberrant peripheral skeletal muscle morphology and function, microcirculatory dysfunction, and dysregulation in muscle mitochondria are implicated in exercise pathophysiology in PAH and contribute to impaired dynamics between microvascular DO_2_, extraction, and utilization reflected in depressed peripheral systemic *EO_2_.* As described earlier, the Fick principle dictates that in the absence of a pulmonary mechanical limitation to exercise, a reduced VO_2_ max can be due to a depressed CO, a depressed C(a-v)O_2_, or both [16]. Impaired *EO_2_* leads to a low C(a-v)O_2_ due to compromised oxygen use at the tissue level, leading to an abnormally high mixed venous oxygen content at peak exercise, which can only be directly ascertained during iCPET.

Impaired peripheral *EO_2_* has previously been described in patients with cardiopulmonary disease including in patients with heart failure with reduced ejection fraction and HFpEF [115], as well as in patients with primary mitochondrial myopathies [116], post-acute sequelae of SARS-CoV-2 infection (PASC) [117], postural tachycardia syndrome (POTS), myalgic encephalomyelitis/chronic fatigue syndrome (ME/CFS) [118], and in a subset of patients with unexplained dyspnea [119]. Data in patients with PAH are limited but provide important insights into disease pathophysiology. In a seminal study, Tolle et al., investigated the systemic extraction ratio (SER) using iCPET, which is the ratio of C(a-v)O_2_ to CaO_2_ during maximum exercise in PAH patients, and found impairments in this variable despite preserved CO and DO_2_ [120]. These findings have been borne out in other studies. For instance, Oliveira et al. showed that patients with resting PH, but not exPH, had evidence of impaired peripheral *EO_2_* at peak exercise. Singh et al. found instead that both those with exPH and PAH had reduced exercise capacity as evidenced by a reduced peak VO_2_ that was driven by a reduced peak CI, likely related to a decreased peak SV index, chronotropic incompetence, and reduced systemic *EO_2_* indicated by a blunted Ca-vO2 difference at peak exercise—the latter supporting the impact of impaired peripheral utilization at the tissue level. Using cardiovascular magnetic resonance augmented niCPET, Brown et al. similarly demonstrated decreased systemic *EO_2_* along with the impaired augmentation of cardiac index in patients with PAH, perhaps related to poor RV contractile reserve [121]. Interestingly, impaired peripheral *EO_2_* has also been described in patients with exPH after treatment with pulmonary vasodilator therapy who experienced a blunting in the expected rise in VO_2_ despite improvement in exercise capacity. This ultimately was attributed to a decrease in the maximum exercise C(a-v)O_2_ posited to be related to the unmasking of systemic microcirculatory dysfunction with PH-directed therapy, suggesting that exPH patients, who perhaps have an early form of PAH, may have distinct physiological concerns when compared with overt PAH patients in relation to the function of their microvasculature [61].

There are a number of possible explanations for this finding of pathologically impaired systemic *EO_2_* in PAH that relate to both convective and diffusion-related O_2_ transport limitations [30]. First, endothelial dysfunction in PAH likely affects systemic circulation beyond the pulmonary vasculature [122,123,124]. This may be due in part to the dysregulation of growth factors and vasoactive mediators including the downregulation of NO and prostacyclin and the upregulation of endothelin-1, thromboxane, and vascular endothelial growth factors, which may lead to local incongruence between perfusion and metabolism in the muscle bed [120,125]. It is possible that systemic inflammation helps to drive this diffuse vasculopathy, illustrated by PAH patients’ high macrophage activation markers and circulating lipopolysaccharide levels, perhaps indicating chronic gut bacterial translocation [126]. Additionally, patients with PAH have been shown to have abnormal circulating endothelial cells (many with higher values of CD36, which is a marker of E-selectin) and an abnormal circulating biomarker profile with increased numbers of circulating endothelial microvesicles, indicating endothelial damage, and a decreased number of circulating progenitor cells, which suggests a decreased capacity for cellular repair [127,128].

Second, impaired *EO_2_* in the PAH population is likely impacted by abnormal peripheral muscle morphology with related aberrant microcirculatory function. Compared to healthy individuals, patients with PAH have been shown to have reduced type I to type II muscle fiber ratio, suggesting possible underlying myopathy and a reliance on glycolytic metabolism [129] that may have an out-of-proportion effect on VO_2_ [130]. It has also been shown that patients with PAH have a reduction in volitional and non-volitional strength of the inspiratory and quadriceps muscles and altered excitation—contraction coupling, which may be related to an upregulation of proteolysis and a downregulation of cell signaling networks that mediate protein synthesis perhaps due to altered levels of pro-inflammatory cytokines, abnormal growth factor induction, and renin–angiotensin system dysfunction [131], as well as a decreased expression of proteins that regulate mitochondrial fusion in muscles and calcium sequestration [132]. Microcirculatory abnormalities in PAH patients are accentuated by peripheral systemic vasoconstriction [133] and lower capillary density. For instance, Malenfant et al. showed that despite normal CO and DO_2_, PAH patients have a decreased skeletal muscle O_2_ supply that is correlated with the degree of capillary rarefaction [134]. This may be due to defects in angiogenesis driven by the downregulation of microRNAs (miRs) such as miR-126, which has been demonstrated in PAH patients with exercise intolerance and demonstrated muscle microvessel loss [135].

Third, it is possible that impaired peripheral EO_2_ in PAH is driven by mitochondrial dysfunction and biogenesis, and patients with PAH have been shown to have a pathogenic shift in glucose metabolism toward glycolysis and abnormalities in fatty acid oxidation, and a number of studies has demonstrated the entwinement of altered pulmonary vascular metabolism in disease pathogenesis [17]. This may also be impacted by processes such as mitochondrial hyperpolarization and strain on the endoplasmic reticulum system, as well as through abnormalities in the mitochondrial structure and number itself [136,137,138]. Finally, it is known that patients with PAH hyperventilate during rest, exercise, and sleep [20]. It is possible that pathologic exercise-related hyperventilation and resultant hypocapnia along with associated ventilatory abnormalities lead to the abnormal offloading of oxygen in the muscle bed due to impaired hemoglobin–oxygen dissociation in the setting of relative alkalemia (i.e., limiting the Bohr effect) [119,139].

### 2.8. Impaired Peripheral EO_2_: Diagnosis and Treatment

On iCPET, PAH patients with impaired peripheral EO_2_ may have variable hemodynamic abnormalities, but in the setting of otherwise well-controlled PVD will have a low aerobic capacity (VO_2_ max <80% predicted) at peak exercise (respiratory exchange ratio > 1.05 and/or peak HR > 85% predicted) with no pulmonary mechanical limitation to exercise. DO_2_ and CO are generally preserved and accompanied by the primary abnormality of an elevated peak mixed venous oxygen saturation and peak venous oxygen content along with a depressed C(a-v)O_2_ and/or C(a-v)O_2_ to hemoglobin ratio. In patients with connective tissue disease without underlying PAH, this has been shown to be associated with a reduced systemic vascular distensibility coefficient α, which contributes to an impairment in tissue oxygen delivery resulting in an earlier AT, but there is not yet evidence that this is the case in patients with PAH [23].

There are no approved treatments for impaired peripheral *EO_2_* in PAH. Therapy options are extrapolated from data from other patient populations including mitochondrial myopathies and ME/CFS, which may not be generalizable to patients with PVD. The most promising approach is supervised exercise therapy, which has been shown to be a safe intervention in well-compensated patients, is associated with a significant improvement in exercise capacity and cardiopulmonary fitness [140], and is recommended as part of a comprehensive treatment strategy in patients with PAH [105]. The rationale for exercise in this population involves the association with improved muscle function, oxidative enzyme activity, and the number of capillaries [141], and the enhancement of endothelial function through arteriolar vasodilation and improvement in NO formation [142]. For patients with comorbid impaired peripheral *EO_2_,* we specifically recommend slowly accelerated exercise training, which has been shown to be beneficial in patients with mitochondrial diseases and POTS [114]. Regarding pharmacologic therapy, there have been no randomized controlled clinical trials in this patient population, though there is currently significant interest in therapies targeting mitochondrial and metabolic dysfunction [136]. Perhaps more interesting to consider is whether available PAH treatments have a secondary effect of improving peripheral *EO_2_*, which may be the case for the newly approved PAH medication, sotatercept. For instance, in a phase 2b study on sotatercept, Waxman et al. showed that treatment with this medication led to improvement in VO_2_ that was attributable to improvement in C(a-v)O_2_, likely due to improved DO_2_ in the setting of the increased hemoglobin levels appreciated with sotatercept treatment [143]. More studies are needed to understand this underlying pathophysiologic feature of PAH and to develop novel therapeutics to target this process to improve patients’ quality of life.

### 2.9. Future Directions in PAH Using iCPET Data

Information gleaned from iCPET data are essential to diagnose early PVD including PAH and to clarify the causes of unexplained dyspnea in this patient population, including that due to impaired peripheral EO_2_. iCPET also provides more nuanced insights into patients’ responses to PAH therapy [143,144] than niCPET and other conventional modalities alone. Beyond this, utilizing iCPET variables both to provide prognostic information and to clarify complex exercise pathophysiology in this patient population, especially in regard to RV-PA coupling interactions, is an area of robust, active research. For instance, iCPET with various adjunctive testing has been used to shed light on the pathophysiologic association between the VE/VCO_2_ slope and altered pulmonary vascular hemodynamics in this disease state [145] and the effects of altered RV contractile reserve during exercise in PAH [146,147]. iCPET has also been utilized to demonstrate an association between PAH disease severity parameters and survival with peak exercise cardiac index [147,148], and between transplant-free survival and the mPAP/CO slope [149]. Additionally, regarding insights into RV-PA coupling, iCPET allows for more physiologic insights into RV afterload that accounts for a more elegant understanding of the contribution of myocardial wall stress, arterial elastance, PAC, and vessel distensibility [150]. In this vein, it has been shown that RV-PA coupling is preserved in patients with stable PAH but reduced in patients with end-stage disease [151], likely due in part to the fact that adequate RV-PA coupling is dependent on preserved PAC [152]. Patients with PAH have also been shown to have a reduced VO_2_ attributed to decreased peak RV stroke work index (RVSWI), a load-dependent measurement of RV contractility in response to PA hemodynamic oscillations and pulsatility, and the effects of depressed PAC including reduced pulmonary vascular reserve load, which shed light on the importance of understanding RV function in relation to the pulsatile components of its afterload [17,54]. Additionally, patients with PAH have been shown to have impaired ventilatory parameters due not only to increased RV afterload but also to RV diastolic stiffness [58]. Other uses of iCPET data include innovative research combining exercise testing with advanced exercise-related multi-omic and metabolic analyses, which has led to findings driving novel approaches to understanding disease pathophysiology and aiding in drug development. These include insights into the relationship between PA glucose metabolism and RV function and PH patient reliance on fatty acid oxidation and nucleotide metabolism during exercise [17,153]; the relationship between tryptophan metabolism and RV function including between kynurenine and RV diastolic function and RV-PA coupling [154]; and the relationship between arginine–NO pathway molecules, catecholamines, and tricarboxylic acid cycle intermediates, and exercise-related RV-PA dysfunction [155].

While the aforementioned data shed important light on PAH pathophysiology, which is undoubtedly moving the needle forward on improving diagnosis and treatment for this vulnerable patient population, more large-scale studies are needed to elucidate the practical application of this information gleaned from iCPET in the clinic and beyond. This is a current priority in the PH academic community and collaborative efforts to better understand aberrant exercise hemodynamics, and their interactions with multi-omic mediators in PVD are underway including the NIH/NHLBI-led Redefining Pulmonary Hypertension through Pulmonary Vascular Disease Phenomics (PVDOMICS) group [156] and the PEX-NET collaboration; additionally, iCPET data are beginning to be incorporated into PAH therapy trial design [143]. With this, an immediate, clinically applicable research goal, which has the promise of moving the field forward, is demonstrating whether exercise hemodynamics provide important prognostic data above that of resting hemodynamics both for the PAH community and beyond.

## 3. Limitations

This review has several limitations. First, the quality of the evidence included is limited by the quality of the studies published on the topics included in this article. Second, while a comprehensive search was performed, it is possible that relevant articles may have been inadvertently excluded. Third, we did not perform a systematic assessment of article quality, and thus it is possible that author bias in article selection and interpretation was introduced. Finally, this review is a narrative review and therefore does not answer hypothesis-driven research questions.

## 4. Conclusions

PAH is a chronic, progressive disease with high morbidity and mortality despite overall improvement in diagnosis, treatment, and supportive care over the past three decades. iCPET facilitates the evaluation of cardiopulmonary hemodynamics that allows for the determination of central and peripheral limitations to exercise. When utilized in PAH patients, iCPET allows for the early diagnosis of disease, including exPH, which ensures the timely initiation of treatment, and is critical for elucidating the underlying mechanisms of dyspnea in ostensibly well-compensated patients. Moving forward, complex physiologic and metabolomic data obtained from iCPET will continue to elucidate the mechanisms of disease pathogenesis in PAH, which has the potential to improve outcomes and health-related quality of life in this patient population.

## Figures and Tables

**Table 1:** Overview of major recent studies (2018–present) exploring prognostic relevance of exercise pulmonary hemodynamics.

Publication/Year	Subjects (n)	Design	Study Population (Inclusion Criteria)	Primary End-Point	Major Prognostically Relevant Hemodynamics Predicting Adverse Events
Kovacs, et al., 2024 [81]	764	Retrospective	Resting mPAP < 25 mmHg, multiple indications for initial RHC (e.g., unexplained dyspnea, elevated PH risk)	All-cause mortality	mPAP/CO slope > 3 mmHg/L/min
Zeder, et al., 2022 [76]	3981	Systematic Review/Meta-analysis	Variable (e.g., HFpEF, pre-capillary PH, unexplained dyspnea)	All-cause mortality +/-heart failure-related hospitalization	mPAP/CO slope > 3 mmHg/L/min PAWP/CO slope > 2 mmHg/L/min
Douschan, et al., 2022 [92]		Retrospective	Resting mPAP < 25 mmHg, suspected PH	All-cause mortality	mPAP/CO slope > 7.5 mmHg/L/min, PAWP/CO slope > 6.0 mmHg/L/min, TPG/CO slope > 3.9 mmHg/L/min, Peak CO < 8.5 L/min
Zeder, et al., 2021 [76]	80	Retrospective	Resting mPAP < 25 mmHg, systemic sclerosis	Time to all-cause mortality	Elevated PVR (>1.8 WU), elevated TPR (>3.3 WU), mPAP/CO slope > 3.5 WU
Ho, et al., 2020 [84]	714	Prospective	LVEF ≥50%, unexplained dyspnea	All-cause mortality	mPAP/CO slope > 3 mmHg/L/min
Eisman, et al., 2018 [83]	175	Retrospective	LVEF > 50% Resting PAWP < 15 mmHg, unexplained dyspnea	Cardiovascular death, abnormal resting PAWP on future RHC, HF hospitalization	PAWP/CO slope > 2 mmHg/L/min
